# Associação entre os Níveis Séricos de Elabela e Oclusão Total Crônica em Pacientes com Angina Pectoris Estável

**DOI:** 10.36660/abc.20200492

**Published:** 2021-09-01

**Authors:** Fethi Yavuz, Mehmet Kaplan

**Affiliations:** 1 Adıyaman University Training and Research Hospital Departamento de Cardiologia Adıyaman Turquia Departamento de Cardiologia, Adıyaman University Training and Research Hospital, Adıyaman - Turquia; 2 Gaziantep University Medicine Faculty Departamento de Cardiologia Gaziantep Turquia Gaziantep University Medicine Faculty, Departamento de Cardiologia, Gaziantep -Turquia

**Keywords:** Oclusão Total Crônica, Oclusão Coronária, Peptídeos, Elabela, Apelina, Angiografia Coronária, Angina Pectoris, Circulação Colateral

## Abstract

**Fundamento:**

Os efeitos benéficos do elabela no sistema cardiovascular foram demonstrados em estudos.

**Objetivo:**

Comparar os níveis séricos de elabela de pacientes com oclusão total crônica (OTC) com pacientes controle com artérias coronárias normais e investigar se há correlação com o desenvolvimento colateral.

**Métodos:**

Estudo transversal e prospectivo. O estudo incluiu cinquenta pacientes (28,0% mulheres, idade média 61,6±7,3 anos) com OTC em pelo menos um vaso coronário e 50 pacientes (38% mulheres, idade média 60,7±6,38 anos) com artérias coronárias normais. Os pacientes do grupo OTC foram divididos em dois grupos: Rentrop 0–1, composto por pacientes com fraco desenvolvimento colateral e Rentrop 2–3, composto por pacientes com bom desenvolvimento colateral. Além da idade, sexo, características demográficas e exames laboratoriais de rotina dos pacientes, foram medidos os níveis de elabela.

**Resultados:**

As características demográficas e os valores laboratoriais mostraram-se semelhantes em ambos os grupos. Ao passo que o nível médio de NT-proBNP e troponina estava maior no grupo OTC, o nível médio de elabela estava menor (p<0,05 para todos). Na análise de regressão multivariada, os níveis de NT-proBNP e elabela foram considerados preditores independentes para OTC. Além disso, o nível de elabela apresentou-se estatisticamente maior em pacientes do grupo Rentrop 2–3 em comparação com os pacientes do grupo Rentrop 0–1 (p<0,05).

**Conclusões:**

Em nosso estudo, mostramos que o nível médio de elabela estava baixo em pacientes com OTC em comparação com pacientes normais. Além disso, constatamos que o nível de elabela é inferior em pacientes com desenvolvimento colateral fraco em comparação com pacientes com bom desenvolvimento colateral.

## Introdução

Define-se oclusão total crônica (OTC) como a obstrução aterosclerótica completa (trombólise em infarto do miocárdio (TIMI) grau 0) do fluxo de artéria coronária, com duração de oclusão estimada de ≥ 3 meses.^1^ Os dados sobre a prevalência da OTC resultam de registros de pacientes submetidos a angiocoronariografia por suspeita de doença arterial coronariana (DAC). Assim, desconhece-se a prevalência geral na população (assintomática). A frequência de pelo menos uma OTC varia de 30% a 50% em pacientes com diagnóstico prévio de DAC submetidos a cateterismo cardíaco.^2^

O elabela foi recentemente descoberto como um novo ligante peptídico endógeno do receptor de apelina (APJ), um receptor acoplado à proteína G. A expressão de elabela é mais elevada no tecido cardíaco embrionário e depois diminui gradualmente. O elabela é encontrado principalmente em fibroblastos e células endoteliais do coração. A sinalização de elabela tem se mostrado crucial para o desenvolvimento normal do sistema cardiovascular durante a embriogênese.^3^ As funções biológicas do elabela no corpo são semelhantes às do apelina, o ligante do outro receptor de apelina. Em geral, o apelina e o elabela são conhecidos por seus efeitos protetores do sistema cardiovascular. A ligação desse receptor e seus ligantes desempenha algumas funções regulatórias no sistema cardiovascular, sistema nervoso central, sistema circulatório e diversos outros sistemas.^4^^,^^5^

O elabela e o apelina promovem a angiogênese por meio do APJ.^6^ O elabela e o apelina desempenham funções em diferentes estágios da morfogênese vascular.^7^ O elabela é mais potente do que o apelina no que diz respeito ao aumento da contratilidade miocárdica e vasodilatação coronariana.^8^ Embora o elabela e o apelina compartilhem muitas semelhanças, eles funcionam por meio de diferentes vias de sinalização e têm diferentes atividades biológicas. No entanto, relativamente pouco se sabe sobre as propriedades e funções biológicas do elabela. Evidências crescentes sugerem que o APJ não é o único receptor do elabela. Sugeriu-se que o elabela possa ter diversos efeitos cardioprotetores por meio de vias adicionais.^9^^,^^10^ Portanto, são necessários outros estudos para elucidar os mecanismos moleculares e as funções biológicas do elabela e a regulação das vias de sinalização celular.

Diversos estudos na literatura examinaram a função do apelina no corpo humano. No entanto, quase todos os estudos acima mencionados sobre o elabela foram realizados in vitro ou em modelos animais. Recentemente, diversas pesquisas em humanos foram relatadas na literatura.^11^^,^^12^ Tanto quanto sabemos, não existem estudos sobre a função do elabela nas doenças cardiovasculares humanas e o seu potencial de tratamento. Em pacientes com OTC, que representa um espectro da doença arterial coronariana, estudos anteriores não investigaram os níveis séricos de elabela em comparação com pacientes com artérias coronárias normais. Tendo em vista que o elabela tem efeitos sobre a angiogênese e arteriogênese, pode ser que haja uma relação entre a extensão do desenvolvimento colateral coronariano e os níveis de elabela. Assim, no presente estudo, nosso objetivo foi investigar os níveis séricos de elabela em pacientes com OTC em comparação com pacientes com artérias coronárias normais e examinar a relação entre o desenvolvimento colateral coronariano e os níveis de elabela.

## Método

### Desenho do estudo

Trata-se de um estudo transversal. A decisão pela angiografia coronária foi feita de acordo com um teste de esforço não invasivo positivo ou alta suspeita clínica de doença arterial coronariana. O tamanho ou poder da amostra não foi calculado para o estudo. Definiu-se o tamanho da amostra por conveniência. O comitê de ética local aprovou o protocolo deste estudo, conduzido de acordo com os princípios estabelecidos na Declaração de Helsinque. Todos os participantes assinaram consentimento informado por escrito antes do início do estudo.

### População do estudo

Cinquenta pacientes com OTC, conforme demonstrado por angiocoronariografia, e 50 pacientes com artérias coronárias normais (grupo controle) foram incluídos no estudo. As características clínicas e demográficas basais da população do estudo, todos os parâmetros laboratoriais de rotina e dados ecocardiográficos foram registrados no formulário especificamente elaborado para este estudo, e gerado para cada paciente. Não houve diferença no tratamento farmacológico entre os pacientes Rentrop 0–1 e 2–3 com OTC.

Definiu-se hipertensão como medidas sistêmicas repetidas da pressão arterial >140/90 mmHg ou uso de medicação anti-hipertensiva. Diagnosticou-se diabetes mellitus de acordo com um dos seguintes critérios: (1) glicemia em jejum ≥126 mg/dL, (2) glicemia >200 mg/dL a qualquer momento, (3) histórico de diabetes mellitus ou pacientes sob medicação antidiabética. Definiu-se hipercolesterolemia como tratamento em curso com hipolipemiantes ou nível basal total de colesterol >200 mg/dL. Definiu-se tabagismo como tabagismo regular nos últimos 6 meses. Definiu-se histórico familiar de doença coronariana como a presença de doença arterial coronariana em parentes de primeiro grau com idade inferior a 55 (masculino) ou 65 (feminino).

Os critérios de exclusão do estudo foram os seguintes: doença cardíaca valvar moderada a grave, doença hepática crônica, doença renal crônica (TFG <60 ml/kg/min), disfunção tireoidiana, fibrilação atrial, síndrome coronariana aguda, malignidade, infecção ativa, doença autoimune, pacientes com idade ≤18 e ≥85.

### Parâmetros laboratoriais

Os parâmetros laboratoriais de rotina (glicose, troponina I de alta sensibilidade, porção N-terminal do pró-hormônio do peptídeo natriurético do tipo B, função renal, painel lipídico e hemograma completo) de todos os pacientes foram analisados. Os níveis séricos de elabela foram obtidos por meio de kits comercialmente disponíveis (Sunred Biological Technology, Xangai, China). Mediu-se a isoforma elabela-32. O kit utiliza um ensaio imunoenzimático (ELISA) sanduíche com duplo anticorpo para determinar o nível de elabela em amostras de sangue. Para medir o apelina, o soro foi separado do sangue por centrifugação a 3.000 rpm por 10 min e mantido congelado a −80 °C até a análise. De acordo com o fabricante, esse ensaio tem um coeficiente de variação interensaio inferior a 12% e um coeficiente de variação intraensaio inferior a 10%.

### Avaliação angiográfica

Realizou-se angiocoronariografia de rotina pela técnica de Judkins com cateteres cardíacos direito e esquerdo de 6 ou 7 French, sem o uso de nitroglicerina, adenosina ou bloqueador dos canais de cálcio. Os angiogramas foram gravados em mídia digital DICOM a 15 quadros/ms e foram revisados por dois angiógrafos experientes que desconheciam o estado clínico dos pacientes. Artérias coronárias normais foram definidas como ausência de lesões coronárias. As OTCs foram vistas a partir de pelo menos dois ângulos após injeção seletiva de material de contraste. Definiu-se OTC como estenose de 100% do diâmetro luminal quando não havia lúmen perceptível e fluxo anterógrado por mais de 3 meses.^1^ As coronariografias foram reavaliadas para circulação colateral por dois cardiologistas intervencionistas experientes completamente cegos para o estudo. A circulação colateral coronariana foi graduada pela classificação Rentrop,^13^ onde grau 0=nenhum preenchimento de vasos colaterais; grau 1=preenchimento de ramos laterais arteriais a serem dilatados por canais colaterais sem visualização do segmento epicárdico; grau 2=preenchimento parcial do segmento epicárdico por vasos colaterais; e grau 3=preenchimento completo do segmento epicárdico da artéria dilatada por vaso colateral. Pacientes com desenvolvimento colateral coronário grau 0‒1 foram considerados como grupo colateral ruim, e pacientes com desenvolvimento colateral coronário grau 2‒3 foram considerados grupo colateral bom.^14^ Em pacientes com mais de 1 vaso colateral preenchendo o vaso ocluído, utilizou-se para análise o vaso colateral com o maior grau Rentrop.

### Análise estatística

Analisou-se a normalidade da distribuição dos dados das variáveis contínuas pelo teste de Kolmogorov-Smirnov. As variáveis com distribuição normal foram apresentadas como média (± desvio padrão) e aquelas sem distribuição normal foram apresentadas como mediana (intervalo interquartil). As variáveis que apresentaram distribuição normal entre os grupos foram comparadas pelo teste *t* de Student não pareado e aquelas sem distribuição normal foram comparadas pelo teste U de Mann-Whitney. As variáveis categóricas foram expressas em números e porcentagens, e comparadas entre os grupos através do teste exato de Fisher ou do qui-quadrado. Foram utilizadas curvas *receiver operating characteristic* (ROC) para demonstrar a sensibilidade e especificidade dos valores de corte do elabela para a OTC. Análises de regressão logística binária foram realizadas para determinar preditores independentes de OTC. Variáveis que apresentavam valor de p<0,05 não ajustado na análise univariada foram incluídas na análise multivariada. As análises estatísticas foram realizadas utilizando o software Statistical Package for Social Science (SPSS 20.0) para Windows (SPSS Inc., Chicago, Illinois, EUA). O valor de p<0,05 foi considerado estatisticamente significativo.

## Resultados

O estudou incluiu cinquenta pacientes com OTC e 50 pacientes com artérias coronárias normais. As características demográficas e clínicas basais dos grupos estudados estão resumidas na Tabela 1. A comparação das características demográficas entre os grupos mostrou que a intervenção coronária percutânea foi mais comumente realizada no grupo OTC (p=0,006). Não houve diferença estatisticamente significativa entre os grupos em termos de idade média, sexo e número de pacientes com hipertensão, diabetes mellitus, hiperlipidemia, doença arterial periférica, insuficiência cardíaca, acidente vascular cerebral e doença pulmonar obstrutiva crônica (todos p>0,05).

**Tabela 1:** Características demográficas basais e fatores de risco cardiovascular da população do estudo

Variável	Pacientes com OTC (n=50)	Controles (-) (n= 50)	p
Idade, anos	61,6 ± 7,31	60,7 ± 6,38	0,495
Sexo feminino, % (n)	28,0 (14)	38 (19)	0,288
Fração de ejeção ventricular esquerda, %	55,3 ± 7,0	55,7 ± 6,83	0,795
Hipertensão, % (n)	70,0 (35)	50 (28)	0,147
Diabetes mellitus, % (n)	66,0 (33)	50 (25)	0,105
Hiperlipidemia, % (n)	62 (31)	44 (22)	0,071
Doença vascular periférica, % (n)	10,0 (5)	6 (3)	0,461
Acidente vascular cerebral, % (n)	4,0 (2)	2(1)	0,558
Insuficiência cardíaca, % (n)	16,0 (8)	12 (6)	0,564
Doença pulmonar obstrutiva crônica, %(n)	10,0 (5)	18 (9)	0,249
Intervenção coronariana percutânea prévia% (n)	14,0 (7)	0 (0)	0,006
Fumante atual, % (n)	58 (29)	48 (24)	0,316
Histórico familiar de DAC, % (n)	22,0 (11)	24 (12)	0,812

A Tabela 2 apresenta os parâmetros laboratoriais e os níveis de elabela. Os níveis médios de NT-proBNP e troponina apresentaram-se significativamente maiores no grupo OTC do que no grupo controle (p<0,05 para ambos). No entanto, os níveis médios de elabela apresentaram-se significativamente mais baixos no grupo OTC do que no grupo controle (p<0,001). Não houve diferenças significativas entre os grupos em relação a outros parâmetros laboratoriais (todos p>0,05).

**Tabela 2:** Parâmetros laboratoriais e níveis de elabela dos grupos do estudo.

Variável	Pacientes com OTC (n=50)	Controles (n=50)	p
Glicemia de jejum, md/dL, mediana (IIQ)	102,5 (28,0)	121 (55,0)	0,057
Hemoglobina, g/dL	13,9 ± 1,90	13,2 ± 1,52	0,107
Hematócrito, %	39,9 ± 5,57	39,3 ± 3,95	0,584
Contagem de glóbulos brancos, x10^3^/mL	8,2 ± 2,02	8,4 ± 2,40	0,692
Contagem de plaquetas, x10^3^/mL	265 ± 82	271 ± 51	0,743
Contagem de neutrófilos, x10^3^/mL	5,4 ± 1,68	5,2 ± 1,57	0,357
Contagem de linfócitos, x10^3^/mL	2,2 ± 0,7	2,1 ± 0,6	0,417
Contagem de monócitos, x10^3^/mL	0,6 ± 0,23	0,7 ± 0,29	0,640
Volume médio plaquetário, fL	8,8 ± 1,14	8,8 ± 1,02	0,933
Colesterol total, mg/dl, mediana (IIQ)	206,5 (53,5)	216 (46,5)	0,955
Colesterol LDL, mg/dL, mediana (IIQ)	132,5 (40,5)	147 (34,5)	0,389
Colesterol HDL, mg/dL	42,1 ± 9,5	44,6 ± 8,5	0,236
Triglicerídeos, mg/dL, mediana (IIQ)	173 (103,7)	159 (101,5)	0,147
Ureia, mg/dL	34,9 ± 13,93	33,4 ±7,89	0,580
Creatinina, mg/dL	0,84 ±0,27	0,78 ±0,15	0,275
Albumina, mg/dL	3,7 ± 0,59	3,8 ± 0,34	0,424
Troponina, ng/mL, mediana (IIQ)	6,0 (12,0)	4,0 (7,0)	0,013
Elabela, ng/mL, mediana (IIQ)	0,64 (0,40)	1,20 (1,14)	<0,001
PCR, mg/L, mediana (IIQ)	1,8 (1,4)	1,4 (2,0)	0,506
NT-proBNP, pg/ml, mediana (IIQ)	25,5 (16,5)	16.0(11,0)	0,001
Escore SYNTAX	12,3 ± 7,1	-	-

*IIQ: Intervalo Interquartil; LDL: Lipoproteína de baixa densidade; HDL: Lipoproteína de alta densidade; PCR: Proteína C-reativa; NT-proBNP: Porção N-terminal do pró-hormônio do peptídeo natriurético do tipo B*.

Em pacientes com OTC, a artéria coronária direita (n=26, 52%) foi o vaso mais afetado, seguida pela artéria descendente anterior esquerda (n=20, 40%), a artéria circunflexa esquerda (n=11, 22%) e múltiplos vasos (n=7, 14%).

**Figura 1:**
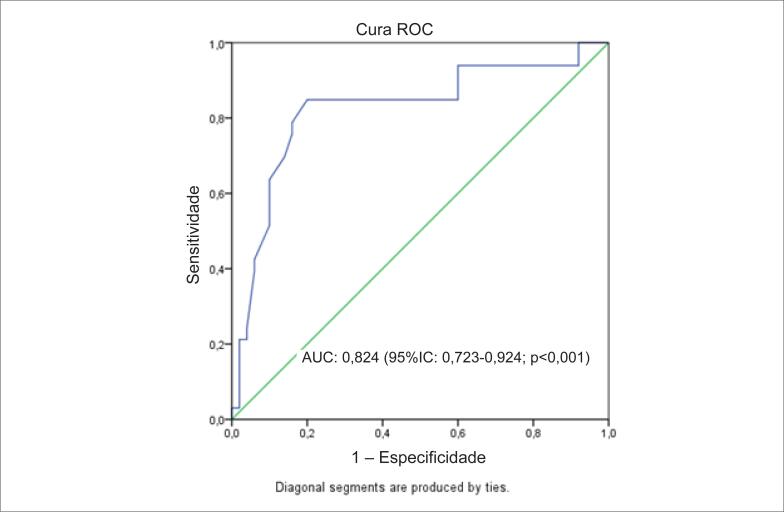
*Análise da curva receiver operating characteristic para o Elabela para predição de OTC*.

Para a previsão de OTC, o valor de corte de <0,9 para elabela apresentou sensibilidade de 84,8% e especificidade de 80,0% (AUC: 0,824; intervalo de confiança de 95%, 0,723–0,924; p<0,001) na análise da curva ROC (Figura 1).

**Figura 2:**
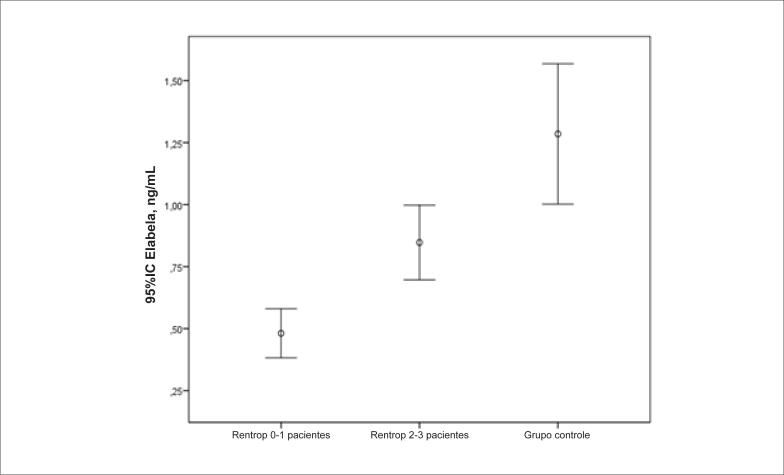
*Relação entre o nível de Elabela e o escore Rentrop (barras de erro: Intervalo de confiança [IC] de 95%)*.

Os pacientes foram divididos em dois grupos de acordo com a classificação Rentrop: Rentrop 0–1 (n=17) e Rentrop 2–3 (n=33). O valor elabela mediano do grupo Rentrop 0–1 (mediana 0,430, IIQ: 0,170) com desenvolvimento colateral ruim foi menor do que o do grupo Rentrop 2–3 (mediana 0,740, IIQ: 0,380) com bom desenvolvimento colateral (p<0,001) (Figura 2).

Os resultados das análises de regressão univariada e multivariada estão resumidos na Tabela III. A análise univariada mostrou que o NT-proBNP, elabela e troponina foram preditores independentes de OTC. No entanto, na análise multivariada, apenas o NT-proBNP (p=0,003) e o elabela (p=0,001) permaneceram como preditores independentes de OTC.

**Tabela 3:** Análises de regressão logística univariada e multivariada

Análise	Univariada		Multivariada	
Variáveis	p	OR [IC de 95%]	p	OR [IC de 95%]
NT-proBNP	0,008	1,071 (1,018- 1,127)	0,003	1,134 (1,043-1.232)
Troponina	0,027	1,100 (1,011-1.197)	-	-
Elabela	<0,001	0,160 (0,112-0.401)	0,001	0.137(0,105-0.358)
Hipertensão	0,149	0,545 (0,239-1.242)	-	-
Diabetes melitus	0,107	0,515 (0,230-1.153)	-	-
Hiperlipidemia	0,073	0,482 (0,217-1.070)	-	-

*NT-proBNP: Porção N-terminal do pró-hormônio do peptídeo natriurético do tipo B; IC: intervalo de confiança*.

## Discussão

O principal achado do nosso estudo foi que os pacientes com OTC apresentaram níveis séricos de elabela mais baixos em comparação com os pacientes com artérias coronárias normais. Além disso, os pacientes com bom desenvolvimento colateral coronariano apresentaram níveis séricos de elabela mais elevados do que pacientes com baixo desenvolvimento colateral coronariano. Até onde sabemos, nosso estudo é o primeiro a examinar a relação entre os níveis séricos de elabela e a OTC e o desenvolvimento colateral coronariano.

O sistema cardiovascular humano contém uma família de peptídeos denominada sistema apelinérgico, composta por receptores de apelina, elabela e APJ.^15^ Estudos têm demonstrado que o apelina e o sistema receptor têm alguns efeitos positivos na aterosclerose, infarto do miocárdio (IM), insuficiência cardíaca e hipertensão arterial pulmonar.^16^^–^^18^ Embora o apelina fosse inicialmente considerado o único ligante para APJ, um novo peptídeo denominado elabela foi identificado por pesquisas conduzidas pela equipe do laboratório Reversade.^19^ Estudos revelaram que o apelina e o elabela agem via APJ e têm funções semelhantes. Além disso, o elabela demonstrou ser mais potente do que o apelina na produção de efeitos cardíacos positivos.^20^ Pacientes com angina estável apresentaram níveis plasmáticos de apelina significativamente mais baixos em comparação com os controles. Além disso, o nível de apelina plasmática esteve negativamente correlacionado com o escore de Gensini em pacientes com síndrome coronariana aguda.^21^ Em seu estudo, Weir et al. mostraram que a concentração plasmática de apelina mostrou-se reduzida no período inicial após o infarto agudo do miocárdio, aumentou significativamente ao longo do tempo, mas permaneceu reduzida em 24 semanas.^22^ Como a OTC é um evento coronariano tardio, o nível de elabela mostrou-se similarmente menor em pacientes com OTC do que em pacientes com artérias coronárias normais.

A OTC é um subgrupo de doença cardíaca aterosclerótica e está associada a desfechos clínicos negativos. As funções biológicas do elabela em doenças ateroscleróticas foram exploradas recentemente em um estudo recente de Li et al., que propôs o elabela como um possível agente terapêutico para doenças cardiovasculares.^23^ O comprometimento da função endotelial está associado à aterosclerose. O elabela é detectado principalmente em fibroblastos no coração e em células endoteliais intactas.^3^ Portanto, a produção de elabela diminui em casos de comprometimento da função endotelial. Em nosso estudo, os baixos níveis de elabela verificados no grupo OTC podem ser explicados pela presença de comprometimento da função endotelial.

O desenvolvimento de colaterais coronários é uma resposta à isquemia miocárdica crônica. Os vasos colaterais fornecem um suprimento sanguíneo alternativo para o miocárdio em pacientes com lesões coronárias obstrutivas. O aumento do fluxo sanguíneo colateral coronário pode reduzir os sintomas de angina e eventos cardiovasculares e manter a função contrátil do miocárdio. Existem evidências importantes de que o desenvolvimento circulatório colateral ocorre como resultado da angiogênese e/ou arteriogênese.^24^ A sinalização do elabela demonstrou ter um papel fundamental no desenvolvimento do sistema cardiovascular normal durante a embriogênese. O elabela promove angiogênese e arteriogênese via APJ.^6^^,^^25^ Em seu estudo, Sharma et al. verificaram que o eixo de sinal elabela-APJ induz a migração de células progenitoras do seio venoso em camundongos e, portanto, desempenha um papel no desenvolvimento dos vasos coronários. Além disso, detectaram defeitos no desenvolvimento de vasos coronários em camundongos sem o sinal elabela-APJ.^26^ Em nosso estudo, o alto nível sérico do elabela em pacientes da classe Rentrop 2–3 com bom desenvolvimento colateral em comparação com pacientes da classe Rentrop 0–1 com desenvolvimento colateral ruim pode ser explicado pelos efeitos positivos do elabela na angiogênese e arteriogênese.

Perjes et al. mostraram em camundongos que os níveis de elabela aumentaram no período pós-IM devido aos seus efeitos cardioprotetores, mas diminuíram posteriormente ou após tratamento malsucedido.^27^ A OTC, um espectro de doença cardíaca aterosclerótica, é definida como estenose de 100% do diâmetro do lúmen por mais de 3 meses, quando não há lúmen perceptível e fluxo anterógrado.^1^ O baixo nível do elabela em pacientes com OTC, conforme encontrado em nosso estudo, é compatível com os achados deste estudo.

Estudos anteriores relataram que o elabela está envolvido em diversos quadros e doenças cardiovasculares. A atividade do sistema apelinérgico aumenta consideravelmente na remodelação após o infarto do miocárdio, na hipertensão arterial, e exerce efeitos benéficos pelo bloqueio do sistema renina-angiotensina-aldosterona^4^. Além disso, os efeitos positivos do elabela na insuficiência cardíaca e na diabetes também foram demonstrados em estudos.^19^^,^^28^^,^^29^ Em nosso estudo, não houve diferença entre os grupos em termos de hipertensão, diabetes e insuficiência cardíaca.

As evidências atuais dos efeitos cardioprotetores do elabela são escassas. No entanto, estudos in vitro e em animais sugerem que o elabela ou um derivado sintético pode ter potencial terapêutico ou biomarcador para doenças cardiovasculares.

### Limitações

O desenho transversal e o tamanho da amostra do estudo são as principais limitações do nosso estudo. Embora medições bioquímicas tenham sido realizadas em nosso estudo, não examinamos o nível do receptor APJ das amostras de tecido. Além disso, não foram obtidas medidas de apelina e APJ, que poderiam ter fornecido mais informações sobre o sistema apelinérgico em pacientes com OTC.

## Conclusão

Neste estudo, mostramos níveis reduzidos de elabela em pacientes com OTC. Também observamos uma correlação positiva entre o desenvolvimento colateral coronariano e os níveis séricos de elabela. São necessários estudos de maiores dimensões e mais abrangentes para estabelecer a função do elabela como um biomarcador cardíaco ou agente terapêutico em humanos.
