# Supplementary material for: Associação entre os Níveis Séricos de Elabela e Oclusão Total Crônica em Pacientes com Angina Pectoris Estável
Source: Arq Bras Cardiol. 2021 Sep 1;117(3):503–10. [Article in Portuguese] doi: 10.36660/abc.20200492 (PMC8462951; doi:10.36660/abc.20200492)
Supplement: Translation

# Translation: Association Between Serum Elabela Levels and Chronic Totally Occlusion in Patients with Stable Angina Pectoris

**Authors:** Fethi Yavuz, Mehmet Kaplan

**Affiliations:** 1 Adıyaman Turkey Departamento de Cardiologia, Adıyaman University Training and Research Hospital, Adıyaman - Turkey; 2 Gaziantep Turkey Gaziantep University Medicine Faculty, Departamento de Cardiologia, Gaziantep -Turkey

**Keywords:** Chronic Total Occlusion, Coronary Occlusion, Peptides, Elabela, Apelin, Coronary Angiography, Angina Pectoris, Colateral Circulation

## Abstract

**Background:**

The beneficial effects of Elabela on the cardiovascular system have been shown in studies.

**Objective:**

To compare serum Elabela levels of chronic total occlusion (CTO) patients with control patients with normal coronary arteries, and to investigate whether there is a correlation with collateral development.

**Methods:**

The study was planned cross-sectionally and prospectively. Fifty patients (28.0% female, mean age 61.6±7.3years) with CTO in at least one coronary vessel and 50 patients (38% female, mean age 60,7±6.38 years) with normal coronary arteries were included in the study. Patients in the CTO group were divided into two groups as Rentrop 0-1, those with weak collateral development, and Rentrop 2-3 with good collateral development. In addition to the age, sex, demographic characteristics and routine laboratory tests of the patients, Elabela levels were measured.

**Results:**

Demographic characteristics and laboratory values were similar in both groups. While the mean NT-proBNP and troponin were higher in the CTO group, the Elabela mean was lower (p <0.05 for all). In the multivariate regression analysis, NT-proBNP and Elabela levels were found to be independent predictors for CTO. Also, Elabela level was found to be statistically higher in Rentrop class 2-3 patients compared to Rentrop class 0-1 patients (p<0.05).

**Conclusion:**

In our study, we showed that the average Elabela level was low in CTO patients compared to normal patients. In addition, we found the level of Elabela to be lower in patients with weak collateral development compared to patients with good collateral development.

## Introduction

Chronic Total Occlusion (CTO) is defined as an atherosclerotic complete obstruction (Thrombolysis in Myocardial Infarction (TIMI) grade 0) of flow of a coronary artery, with an estimated occlusion duration of ≥ 3 months.^1^ Data on the prevalence of CTO are derived from registries of patients undergoing coronary angiography for suspected coronary artery disease (CAD); thus, the overall prevalence in the general (asymptomatic) population is unknown. The frequency of at least one CTO ranges from 30% to 50% in patients with a prior diagnosis of CAD who undergo cardiac catheterization.^2^

Elabela was recently discovered as a new endogenous peptidic ligand of the Apelin receptor (APJ), a G protein-coupled receptor. Elabela expression is highest in embryonic heart tissue and then declines gradually. Elabela is mainly found in fibroblasts and endothelial cells in the heart. Elabela signaling has been demonstrated to be crucial for normal development of the cardiovascular system during embryogenesis.^3^ The biological functions of Elabela in the body are similar to those of Apelin, the ligand of the other apelin receptor. In general, Apelin and Elabela are known for their protective effects on the cardiovascular system. Binding of this receptor and its ligands plays some regulatory roles in the cardiovascular system, central nervous system, circulatory system and many other systems.^4^^,^^5^

Elabela and Apelin promote angiogenesis through APJ.^6^ Elabela and Apelin play roles in different stages of vascular morphogenesis.^7^ Elabela is more potent than Apelin when it comes to increasing myocardial contractility and coronary vasodilation.^8^ Although Elabela and Apelin share many similarities they function through different signaling pathways and have different biological activities. However, relatively little is known about the biological properties and functions of Elabela. Mounting evidence suggests that APJ is not the only receptor for Elabela. It was suggested that Elabela may have multiple cardioprotective effects via additional pathways.^9^^,^^10^ Therefore, further studies are warranted to elucidate the molecular mechanisms and biological roles of Elabela and the regulation of cellular signaling pathways.

There are several studies in the literature that examined the role of Apelin in the human body. However, almost all of the aforementioned studies on Elabela have been conducted in vitro or in animal models. Recently, a number of research studies in humans have been reported r in the literature.^11^^,^^12^ To our best knowledge, there is no study on the role of Elabela in human cardiovascular disease and its treatment potential. In patients with CTO, which represents a spectrum of coronary artery disease, former studies did not investigate serum Elabela levels compared to patients with normal coronary arteries. Since Elabela has effects on angiogenesis and arteriogenesis, there may be a relationship between the extent of coronary collateral development and Elabela levels. Thus, in the present study, we aimed to investigate serum Elabela levels of patients with CTO in comparison to patients with normal coronary arteries and to examine the relationship between coronary collateral development and Elabela levels.

## Method

### Study design

This was a cross-sectional study. The decision for coronary angiography was made according to a positive noninvasive stress test or high clinical suspicion for a coronary artery disease. Sample size or power was not calculated for the study. The sample size was defined for convenience. The local ethics committee approved the study protocol which was implemented in accordance with the principles established in the Declaration of Helsinki. All subjects signed written informed consent prior to initiation of the study.

### Study population

Fifty patients with CTO as demonstrated by coronary angiography and 50 patients with normal coronary arteries (control group) were included in the study. Baseline clinical and demographic characteristics of the study population, all routine laboratory parameters, and echocardiographic data were recorded in the study-specific forms that were generated for each patient. There was no difference in pharmacological treatment between Rentrop Class 0-1 and 2-3 CTO patients.

Hypertension was defined as >140/90 mmHg in repeated systemic blood pressure measurements or use of antihypertensive drugs. Diabetes mellitus was defined according to the presence of one of the following criteria: (1) fasting blood glucose ≥126 mg / dL, (2) blood glucose > 200 mg / dL at any time, (3) a history of diabetes mellitus, or anti-diabetic medication use. Hypercholesterolemia was defined as current treatment with lipid-lowering agents or a baseline total cholesterol level >200 mg/dL. Smoking was defined as regular smoking for the past 6 months. Family history of coronary artery disease was defined as the presence of coronary artery disease in first-degree relatives of males under 55 years of age or females under 65 years of age.

The study exclusion criteria were as follows: known moderate to severe valvular heart disease, chronic liver disease, chronic kidney disease (GFR <60 ml/kg/min), thyroid dysfunction, atrial fibrillation, acute coronary syndrome, malignancy, active infection, autoimmune disease, patients age ≤ 18 and ≥ 85 years.

### Laboratory parameters

Routine laboratory parameters (glucose, high-sensitivity troponin I, N-terminal pro-brain natriuretic peptide, renal function, lipid panel and complete blood count) were analyzed for all patients. Serum Elabela levels were obtained using commercially available kits (Sunred Biological Technology, Shanghai, China). Elabela-32 isoform was measured. The kit uses a double-antibody sandwich enzyme-linked immunosorbent assay (ELISA) to determine the level of Elabela in blood samples. To measure apelin, serum was separated from the blood by centrifugation at 3,000 rpm for 10 min and kept frozen at −80°C until analysis. According to the manufacturer, this assay has an inter-assay coefficient of variation of less than 12% and an intra-assay coefficient of variation of less than 10%.

### Angiographic evaluation

Coronary angiography was routinely performed using the Judkins technique with 6- or 7-French right and left heart catheters without the use of nitroglycerin, adenosine, or a calcium channel blocker. Angiograms were recorded in DICOM format at 15 frames/sec and were reviewed by two experienced angiographers who had no knowledge of the patients' clinical condition. Normal coronary arteries were defined as the absence of coronary lesions. CTOs were visualized from at least two angles after selective injection of the contrast material. CTO was defined as a 100% stenosis of the luminal diameter when there was no noticeable lumen and anterograde flow for more than 3 months.^1^ Coronary angiograms were re-evaluated for collateral circulation by two experienced interventional cardiologists who were completely blinded to the study. The coronary collateral circulation was graded using the Rentrop classification,^13^ where grade 0=no filling of collateral vessels; grade 1=filling of side branches of the artery to be dilated via collateral channels without visualization of the epicardial segment; grade 2=partial filling of the epicardial segment by collateral vessels; and grade 3=complete filling of the epicardial segment of the artery being dilated by a collateral vessel. Patients with grade 0–1 coronary collateral development were considered as poor collateral group, and patients with grade 2–3 coronary collateral development were considered as good collateral group.^14^ In patients with more than 1 collateral vessel filling the occluded vessel, the collateral vessel with the highest Rentrop grade was used for analysis.

### Statistical analysis

Continuous variables were analyzed for data normality using the Kolmogorov-Smirnov test. The variables following a normal distribution were presented as mean (± standard deviation) and those without a normal distribution were presented as median (interquartile range). The variables that showed normal distribution between the groups were compared using the unpaired Student's t-test and those without a normal distribution were compared using the Mann-Whitney U-test. Categorical variables were summarized as numbers and percentages, and compared between the groups using the Chi-square test or the Fisher's Exact test. The receiver operating characteristics (ROC) curve was used to demonstrate the sensitivity and specificity of Elabela cut-off values for CTO. Binary logistic regression analyses were performed to determine independent predictors of CTO. Variables with an unadjusted p value <0.05 in univariate analysis were included in the multivariate analysis. All statistical analyses were performed using the Statistical Package for the Social Sciences (SPSS 20.0) for Windows (SPSS Inc., Chicago, Illinois, USA). A p value <0.05 was considered statistically significant.

## Results

A total of 50 CTO patients and 50 patients with normal coronary arteries were included in the study. Baseline demographic and clinical characteristics of the study groups are summarized in Table 1. Comparison of the demographic features between groups showed that previous percutaneous coronary intervention was more commonly performed in the CTO group (p=0.006). There was no statistically significant difference between groups in terms of mean age, sex, and the number of patients with hypertension, diabetes mellitus, hyperlipidemia, peripheral artery disease, heart failure, stroke, and chronic obstructive pulmonary disease (all p>0.05).

**Table 1:** Baseline demographic characteristics and cardiovascular risk factors of the study population

Variable	CTO patients (n=50)	Controls (-) (n= 50)	p
Age, years	61.6 ± 7.31	60,7 ± 6.38	0.495
Female, % (n)	28.0 (14)	38 (19)	0.288
Left ventricular ejection fraction, %	55.3 ± 7.0	55.7 ± 6.83	0.795
Hypertension, % (n)	70.0 (35)	50 (28)	0.147
Diabetes mellitus, % (n)	66.0 (33)	50 (25)	0.105
Hyperlipidemia, % (n)	62 (31)	44 (22)	0.071
Peripheral vascular disease, % (n)	10.0 (5)	6 (3)	0.461
Stroke, % (n)	4.0 (2)	2(1)	0.558
Heart failure, % (n)	16.0 (8)	12 (6)	0.564
Chronic obstructive pulmonary disease, %(n)	10.0 (5)	18 (9)	0.249
Previous Percutaneous Coronary Intervention% (n)	14.0 (7)	0 (0)	0.006
Current smoker, % (n)	58 (29)	48 (24)	0.316
Family history of CAD, % (n)	22.0 (11)	24 (12)	0.812

*CAD: Coronary artery disease*

Laboratory parameters and Elabela levels are presented in Table 2. Mean NT-proBNP and troponin levels were significantly higher in the CTO group than in the control subjects (p<0.05, for both). However, mean Elabela levels were significantly lower in the CTO group than in control subjects (p <0.001). There were no significant differences between groups with respect to other laboratory parameters (all p>0.05).

**Table 2:** Laboratory parameters and Elabela levels of the study groups.

Variable	CTO patients (n=50)	Controls (n= 50)	P
Fasting glucose level, mg/dL, median (IQR)	102.5 (28.0)	121 (55.0)	0.057
Hemoglobin, g/dL	13.9 ± 1.90	13.2 ± 1.52	0.107
Hematocrit, %	39.9 ± 5.57	39.3 ± 3.95	0.584
White blood cell count, x10^3^/mL	8.2 ± 2.02	8.4 ± 2.40	0.692
Platelet count, x10^3^/ mL	265 ± 82	271 ± 51	0.743
Neutrophil count, x10^3^/ mL	5.4 ± 1.68	5.2 ± 1.57	0.357
Lymphocyte count, x10^3^/ mL	2.2 ± 0.7	2.1 ± 0.6	0.417
Monocyte count, x10^3^/ mL	0.6 ± 0.23	0.7 ± 0.29	0.640
Mean platelet volume, fL	8.8 ± 1.14	8.8 ± 1.02	0.933
Total cholesterol, mg/dL, median (IQR)	206.5 (53.5)	216 (46.5)	0.955
LDL-Cholesterol, mg/dL, median (IQR)	132.5 (40.5)	147 (34.5)	0.389
HDL-Cholesterol, mg/ dL	42.1 ± 9.5	44.6 ± 8.5	0.236
Triglyceride, mg/dL, median (IQR)	173 (103.7)	159 (101.5)	0.147
Urea, mg/dL	34.9 ± 13.93	33.4 ±7.89	0.580
Creatine, mg/dL,	0.84 ±0.27	0.78 ±0.15	0.275
Albumin, mg/dL	3.7 ± 0.59	3.8 ± 0.34	0.424
Troponin, ng/ml median (IQR)	6.0 (12.0)	4.0 (7.0)	0.013
Elabela, ng/mL, median (IQR)	0.64 (0.40)	1.20 (1.14)	<0.001
CRP, mg/l, median (IQR)	1.8 (1.4)	1.4 (2.0)	0.506
NT-proBNP, pg/ml, median (IQR)	25.5 (16.5)	16.0(11.0)	0.001
SYNTAX score	12.3 ± 7.1	-	-

*IQR: Interquartile Range, LDL: Low-density lipoprotein, HDL: High-density lipoprotein*, *CRP: C reactive protein, NT-proBNP: N-terminal pro-brain natriuretic peptide*

In CTO patients, the right coronary artery (n=26, 52%) was the most affected vessel, followed by the left anterior descending artery (n=20, 40%), the left circumflex artery (n=11, 22%) and multiple vessels (n=7, 14%).

For the prediction of CTO, the cut-off value of <0.9 for Elabela had a 84.8% sensitivity and 80.0% specificity (AUC: 0.824; 95% confidence interval, 0.723-0.924; p<0.001) in the ROC curve analysis (Figure 1).

**Figure 1:** *Receiver–operating characteristic curve analysis for Elabela for prediction of CTO*.

The patients were divided into two groups based on the Rentrop classification: Rentrop 0-1 (n=17) and Rentrop 2-3 (n=33). The median Elabela value of the Rentrop 0-1 group (median 0.430, IQR: 0.170) with poor collateral development was lower than that of the Rentrop 2-3 group (median 0.740, IQR: 0.380) with a good collateral development (p<0.001) (Figure 2).

**Figure 2:** *Relationship between Elabela level and Rentrop score (error bars: 95% confidence interval [CI])*.

The results of univariate and multivariate regression analyses are summarized in Table III. Univariate analysis showed that NT-proBNP, Elabela and troponin were independent predictors of CTO. However, in the multivariate analysis, only NT-proBNP (p=0.003) and Elabela (p=0.001) remained as independent predictors of CTO.

## Discussion

The main finding of our study was that CTO patients had lower serum Elabela levels compared to patients with normal coronary arteries. In addition, patients with a good coronary collateral development had higher serum Elabela levels than patients with poor coronary collateral development. To our best knowledge, our study is the first to examine the relationship between serum Elabela levels and CTO and coronary collateral development.

The human cardiovascular system contains a family of peptides called the Apelinergic system, consisting of Apelin, Elabela and APJ receptors.^15^ Studies have shown that Apelin and the receptor system have some positive effects on atherosclerosis, myocardial infarction (MI), heart failure, and pulmonary artery hypertension.^16^^–^^18^ Although Apelin was initially believed to be the only ligand for APJ, a new peptide they named Elabela was identified by the research conducted by the Reversade team.^19^ It has been shown in studies that Apelin and Elabela act via APJ and have similar functions. Moreover, Elabela was shown to be more potent than Apelin in producing positive cardiac effects.^20^ Patients with stable angina were found to have significantly lower plasma Apelin levels in comparison to controls. In addition, plasma Apelin level was found to be negatively correlated with Gensini score in patients with acute coronary syndrome.^21^ In their study, Weir et al. showed that plasma Apelin concentration was reduced in the early period after acute myocardial infarction, increased significantly over time, but remained depressed at 24 weeks.^22^ As CTO is a late coronary event, Elabela level was similarly lower in CTO patients than in patients with normal coronary arteries.

CTO is a subgroup of atherosclerotic heart disease and is associated with negative clinical outcomes. The biological roles of Elabela in atherosclerotic diseases have recently been explored in a recent study by Li et al. who proposed Elabela as a new possible therapeutic agent for cardiovascular diseases.^23^ Impaired endothelial function is associated with atherosclerosis. Elabela is mainly detected in fibroblasts in the heart and intact endothelial cells.^3^ Therefore, Elabela production decreases in impaired endothelial function. In our study, low Elabela levels measured in the CTO group can be explained by the presence of of impaired endothelial function.

The development of coronary collaterals is a response to chronic myocardial ischemia. Collateral vessels provide an alternative blood supply for myocardium in patients with obstructive coronary lesions. Increased coronary collateral blood flow can reduce angina symptoms and cardiovascular events and maintain the contractile function of the myocardium. There is important evidence that collateral circulatory development occurs as a result of angiogenesis and/or arteriogenesis.^24^ Elabela signaling has been shown to have a key role in the development of normal cardiovascular system during embryogenesis. Elabela promotes angiogenesis and arteriogenesis via APJ.^6^^,^^25^ In their study, Sharma et al. found that the Elabela-APJ signal axis induces progenitor cell migration from sinus venosus in mice and thus plays a role in the development of coronary vessels. In addition, they detected defects in coronary vessel development in mice lacking the Elabela-APJ signal.^26^ In our study, the high serum level of Elabela in Rentrop class 2-3 patients with good collateral development compared to patients in Rentrop class 0-1 with poor collateral development can be explained by the positive effects of Elabela on angiogenesis and arteriogenesis.

Perjes et al. showed in mice that Elabela levels increased in the early post- MI period due to its cardioprotective effects but decreased later or after unsuccessful treatment.^27^ CTO, a spectrum of atherosclerotic heart disease, is defined as 100% lumen diameter stenosis for more than 3 months when there is no noticeable lumen and anterograde flow.^1^ The low level of Elabela in CTO patients as found in our study is consistent with the findings of that study

Previous studies reported that Elabela is involved in a wide range of cardiovascular conditions and diseases. Apelinergic system activity increases substantially in remodeling after myocardial infarction, in arterial hypertension and exerts beneficial effects by blocking the Renin-Angiotensin-Aldosterone system^4^. In addition, positive effects of Elabela on heart failure and diabetes have also been shown in studies^19^^,^^28^^,^^29^. In our study, there was no difference between the groups in terms of hypertension, diabetes and heart failure.

Current evidence for the cardioprotective effects of Elabela is scarce. However, in vitro and animal studies suggest that Elabela or a synthetic derivative may have therapeutic or biomarker potential for cardiovascular diseases.

### Limitations

The cross-sectional design and the sample size of the study are the main limitations of our study. Although biochemical measurements were performed in our study, we did not examine the APJ receptor level of the tissue samples. Also, Apelin and APJ measurements were not obtained and they could have provided more information about the Apelinergic system in CTO patients.

## Conclusion

In this study, we showed decreased Elabela levels in CTO patients. We also observed a positive correlation between coronary collateral development and serum Elabela levels. Larger and more comprehensive studies are needed to establish the role of Elabela as a cardiac biomarker or therapeutic agent in humans.
